# New Insights into the Origin and Evolution of Mysmenid Spiders (Araneae, Mysmenidae) Based on the First Four Complete Mitochondrial Genomes

**DOI:** 10.3390/ani13030497

**Published:** 2023-01-31

**Authors:** Shiliang Liu, Shuqiao Wang, Qian Chen, Chuang Zhou, Yucheng Lin

**Affiliations:** 1 Key Laboratory of Bio-Resourses and Eco-Environment (Ministry of Educaion), College of Life Sciences, Sichuan University, Chengdu 610064, China; 2 The Sichuan Key Laboratory for Conservation Biology of Endangered Wildlife, Sichuan University, Chengdu 610064, China

**Keywords:** Mysmenidae, comparative analysis, mitochondrial genome, structure, phylogenetic analyses

## Abstract

**Simple Summary:**

The complete mitochondrial genome has been widely applied in the phylogenetics, population genetics and ecological research of animals due to its characteristics such as strict maternal inheritance and comparatively conserved genomic structure. Currently, the complete mitochondrial genomes of the Mysmenidae are not available. In this study, we reported the first four complete mitochondrial genomes of the Mysmenidae, including one aboveground species (*Trogloneta yuensis*) and three cave-dwelling species (*T. yunnanense*, *Yamaneta kehen* and *Y. paquini*). *T. yunnanense* was more similar to *Yamaneta* in mitogenome size than *T. yuensis*, possibly showing the convergent evolution of cave spiders. High variability was detected between the genera *Trogloneta* and *Yamaneta*. The phylogenetic analysis supports that Mysmenidae is a sister clade to the family Tetragnathidae. Our data and findings could enrich the gene database and contribute to the better understanding of the molecular characteristics of the family Mysmenidae, which will provide help for further studies related to the population genetics, molecular biology and phylogenetics of these spiders.

**Abstract:**

The mitochondrial genome (mitogenome) is recognized as an effective molecular marker for studying molecular evolution and phylogeny. The family Mysmenidae is a group of widely distributed and covert-living spiders, of which the mitogenomic information is largely unclear. In this study, we obtained the first four complete mitogenomes of mysmenid spiders (one aboveground species: *Trogloneta yuensis*, and three cave-dwelling species: *T. yunnanense*, *Yamaneta kehen* and *Y. paquini*). Comparative analyses revealed that their lengths ranged from 13,771 bp (*T. yuensis*) to 14,223 bp (*Y. kehen*), containing a standard set of 37 genes and an A + T-rich region with the same gene orientation as other spider species. The mitogenomic size of *T. yunnanense* was more similar to that of *Yamaneta* mitogenomes than that of *T. yuensis*, which might indicate the convergent evolution of cave spiders. High variability was detected between the genera *Trogloneta* and *Yamaneta*. The A + T content, the amino acid frequency of protein-coding genes (PCGs) and the secondary structures of tRNAs showed large differences. *Yamaneta kehen* and *Y. paquini* contained almost identical truncated tRNAs, and their intergenic spacers and overlaps exhibited high uniformity. The two *Yamaneta* species also possessed a higher similarity of start/stop codons for PCGs than the two *Trogloneta* species. In selective pressure analysis, compared to *Yamaneta*, *Trogloneta* had much higher Ka/Ks values, which implies that selection pressure may be affected by habitat changes. In our study, the phylogenetic analysis based on the combination of 13 PCGs and two rRNAs showed that Mysmenidae is a sister clade to the family Tetragnathidae. Our data and findings will contribute to the better understanding of the origin and evolution of mysmenid spiders.

## 1. Introduction

Spiders are among the most diverse groups dating back to the Late Carboniferous. They are found in almost all terrestrial ecosystems on the planet and considered one of the most successful animal groups due to their outstanding evolutionary radiation and ecological plasticity [1,2]. The order Araneae currently contains more than 50,000 described species in 132 families [3]. The World Spider Catalog has recorded a total of 158 species in 14 genera of Mysmenidae. Although the family Mysmenidae is distributed worldwide, it is one of the least-studied family-level groups among orb-weaving spiders, and its diversity is grossly undersampled due to their small size and cryptic life style [4]. Most previous studies were restricted to a relatively small dataset for phylogenetic analyses with limited morphological and/or behavioral characteristics, or a few mitochondrial/nuclear gene sequences such as cytochrome oxidase subunit 1 (cox1), rRNAs (16S and 12S) and tRNAs, and nuclear genes of rDNA (18S and 28S) and histone (H3) [4,5,6]. These relatively short and rapidly evolving genetic makers are generally inadequate for resolving deeper-level relationships [7].

Generally, a typical mitochondrial genome (mitogenome) of a metazoan is a closed-circular, double-stranded molecule of 11–20 kb in size, which contains 13 protein-coding genes (PCGs), 22 transfer RNAs (tRNAs), two ribosomal RNAs (16S and 12S rRNA) and a non-coding sequence known as the control region for replication and transcription [1]. Since the 1980s, the mitogenome has given a new impetus to evolutionary genetics [8], which is characterized by a rapid evolutionary rate, a low recombination rate, maternal inheritance, a neutral evolution pattern and haploidy [9]. For example, birds and crocodiles are closely related in evolutionary history [10], and hippopotamus and whales began to diverge from about 55 million years ago [11].

Mysmenidae is widely distributed in tropical and temperate regions, even in some extreme environment, such as caves [4]. A cave is a special ecosystem with no sunlight at all, no plant growth, a high CO_2_ concentration, a constant temperature that is close to the mean annual region temperature and little food [12]. To adapt to these hard habitats, the cave-dwelling species exhibit diverse morphological characteristics such as pigment reduction, eye regression and appendage elongation [13]. *Trogloneta yunnanense* [14], *Yamaneta kehen* and *Y. paquini* [15] are karst cave spiders that live in the mountains of southwest China. The unavailability of complete mitogenomes of these cave-dwelling spiders could definitely confine the understanding of the evolution and genetic adaptation of these troglobionts. In this study, we sequenced, annotated and characterized the first four spider mitogenomes of the Mysmenidae (the three cave species aforementioned and Trogloneta yuensis, an aboveground species), to (1) explore the general characterizations of Mysmenidae mitogenomes, (2) assess whether the selection pressure between different habitats exhibits some differences and (3) further conduct phylogenetic analysis to explore the place of Mysmenidae in the evolutionary history of Araneae.

## 2. Materials and Methods

### 2.1. Sampling

The samples of four spider species were collected from Yunnan, Guizhou and Hunan Provinces, China, and their sampling locality information is provided in Table 1. The field collections did not involve endangered or protected species, and no specific permits were required for our collecting. Each specimen was preserved in the field in 95% ethanol and taken back to the laboratory stored at −20 °C. We identified species by the morphology of male palp and female epigyne.

### 2.2. DNA Extraction, Sample Preparation and Genome Sequencing

Total genomic DNA was extracted from the legs and prosomatic tissues of spider samples using the DNeasy Blood and Tissue Kit (Qiagen; P/N: 69506) (Hilden, NRW, Germany). Mitochondrial genome sequences were generated using NovaSeq 6000 (Illumina, San Diego, CA, USA) with paired reads of 2 × 150 bp in Tsingke Biotechnology Co., Ltd. (Beijing, China).

### 2.3. Mitogenome Assembly, Annotation, Visualization and Comparative Analysis

The overall quality of the sequences was assessed from their Phred scores using fastp software [16]. Ambiguous nucleotides and raw sequence reads with lower than Q20 Phred score were trimmed and removed. NOVOPlasty [17] was used to de novo assemble the mitogenome. The assembled mitogenomes were submitted to MITOS WebServer [18] for initial gene annotation. The regions of protein-coding genes (PCGs), transfer RNAs (tRNAs) and ribosomal RNAs (rRNAs) were further validated using nucleotide–nucleotide BLAST (BLASTn) [19]. The maps of the mitogenomes were drawn using OGDraw v1.2 software (http://ogdraw.mpimp-golm.mpg.de/) (accessed on 20 March 2022) [20].

The secondary structures of 22 tRNA genes were predicted by MITOS WebServer [18], tRNAscan-SE 2.0 WebServer [21] and ARWEN [22]. For those tRNA genes failed to be identified, we determined them by comparing the mitogenomes with published spider mitogenomes in GenBank and proofread tRNA secondary structure features.

Statistical analyses of base content, amino acid composition and codon usage in the mitogenome sequences of four spider species were performed using MEGA X [23]. Overlapping regions and spacer regions between genes were detected manually. The mitochondrial genome skew values were calculated as follows: AT skew = (A − T)/(A + T) and GC skew = (G − C)/(G + C) [24]. The non-synonymous substitution rate (Ka), synonymous substitution rate (Ks) and Ka/Ks ratio were inferred with DnaSP v6.0 (the Ka/Ks ratio >1, =1, and <1 indicate positive, neutral and purifying selection, respectively) [25].

### 2.4. Phylogenetic Analysis

A total of 55 mitogenomes available in the Genebank and four mitogenomes obtained in this study were used for phylogenetic analysis, including 58 mitogenomes of spider species and a mitogenome of *Limulus polyphemus* (Table 2). *Limulus polyphemus* is an ancient and slow morphological-evolving group of species, and is regarded as a keystone group for studies of evolution and arthropod phylogeny [26]. It was used as outgroup in this analysis. We aligned each gene of 13 PCGs and two rRNAs respectively, and Gblock [27] was used to analyze them, and then concatenated them all. The final aligned sequences were 10,506 sites for 13 PCGs and 1378 sites for two rRNAs. We used DAMBE to test whether the sequence is suitable for constructing a phylogenetic tree [28]. ModelFinder was used to find the most suitable model for both Maximum Likelihood (ML) analysis and Bayesian inference (BI) under the Akaike information criterion (AIC) [29]. The BI analysis was performed by using Mrbayes 3.2.7a [30] with two simultaneous runs and four Monte Carlo Markov chains (10,000,000 generations, sampling every 1000 generations and the first 25% of sampled trees were burn-in) until the average standard deviation of split frequencies was less than 0.01. The ML phylogenetic analysis was conducted in IQ-TREE v1.6.12 [31] with 1000 standard bootstrap replicates. In this study, ML and BI trees were constructed by a set of software integrated in the PhyloSuite program [32].

## 3. Results and Discussion

### 3.1. Mitogenome Features

**Figure 1:**
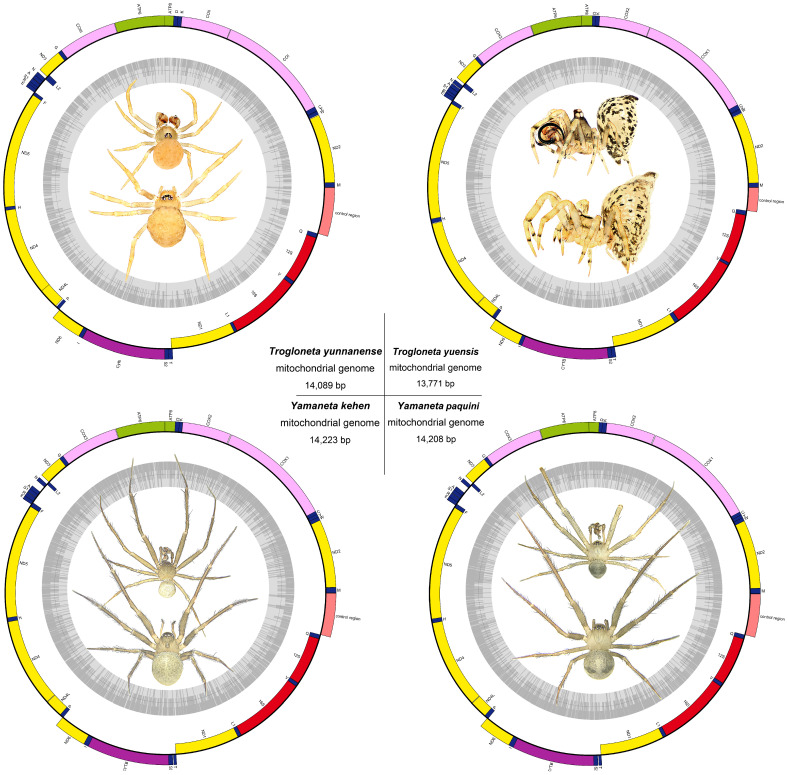
Circular maps of the mitogenomes of four Mysmenidae species. Protein-coding and ribosomal genes are shown with standard abbreviations.

We finally obtained 43,486,268 clean reads in *Trogloneta yunnanense*, 35,901,936 clean reads in *Trogloneta yuensis*, 39,136,196 clean reads in *Yamaneta kehen* and 38,382,448 clean reads in *Yamaneta paquini*, Each of the new complete mitogenomes of *T. yunnanense*, *T. yuensis*, *Y. Kehen* and *Y. paquini* could be circularized, and the total lengths of the four circular complete mitogenomes were 14,089 bp, 13,771 bp, 14,223 bp and 14,208 bp, respectively (Table 3; Figure 1). They were relatively smaller than other spider mitogenomes [33], especially *T. yuensis*, owing to its tiny A + T-rich region and extremely truncated tRNAs. A previous study on *Habronattus oregonensis* of the Salticidae indicated an overall trend toward minimization of the spider mitogenomes [33]. Interestingly, the mitogenome size of *T. yunnanense* was more similar to that of *Yamaneta* than *T. yuensis*. Each mitogenome shared the same 37 typical metazoan genes (13 PCGs, 22 tRNAs and two rRNA genes,) and a non-coding control region (Table 3). Meanwhile, the gene order of these four mitogenomes was conserved and identical to many other spiders. Circular maps of all four mitogenomes are shown in Figure 1. Similar to other published spider mitogenomes, the nucleotide composition of the mitochondrial genomes of the four spiders clearly favored A/T of the J-strands (Table 4). The A + T content of *Trogloneta* was significantly higher than *Yamaneta* in each mitogenome region (Table 4). Additionally, all four mitogenomes showed negative AT-skews (–0.174 to –0.054) and positive GC-skews (0.288 to 0.428) (Table 4). The AT-skews of the genus *Trogloneta* were lower than those of the genus *Yamaneta*, while the GC-skews of *T. yunnanense*, *Y. kehen* and *Y. paquini* were similar but not for *T. yuensis* (Table 3).

Each mitogenome had a large number of intergenic sequences (spacers and overlaps) (Table 2). Mitogenomes of these four spiders were characterized with more intergenic overlaps than spacers. The largest spacer of *Yamaneta* was located between *trnN* and *trnA* (89 bp in *Y. kehen* and 87 bp in *Y. paquini*), which were much longer than those of *Trogloneta* (both 10 bp between *trnP* and *nad6*) (Table 2). The higher content of intergenic overlaps than spacers was also evident in the mitogenomes of other spider species: such as *Tetragnatha maxillosa*, *Tetragnatha nitens*, *Neoscona Scylla* and *Lyrognathus crotalus* [34,35,36]. Moreover, the spacers and overlaps of *Yamaneta* showed higher uniformity than those of *Trogloneta*.

### 3.2. Protein-Coding Genes and Codon Usage

Among the 13 PCGs, only four genes (*nad4*, *nad4L*, *nad5* and *nad1*) were encoded on the minority strand (N-strand), while the others were encoded on the majority strand (J-strand). In *Trogloneta,* the start codons of PCGs were characterized with five types: ATA, ATT, ATG, TTA and TTG, while there were six codon types in *Yamaneta* (additionally including ATC) (Table 2). Most PCGs terminated with the TAA or TAG stop codon, while *nad4* and *nad4L* had an incomplete stop codon T−.

The mitogenomes of *Trogloneta* only possessed five pairs of identical start/stop codons, while the *Yamaneta* had *cox1* (TTA/TAA), *atp6* (ATG/TAA), *nad4* (ATA/T), *nad4L* (ATT/T) and *nad5* (ATA/TAA) (Table 3). Overall, the presence of ATN as start codons was common in most PCGs except for *cox1*, *cox2*, *cox3* and *nad6*. The start codons for *cox1* and *cox3* were TAA and TTG in these four spider species, respectively. The start codon for *cox2* was TTG in *Trogloneta* and ATT in *Yamaneta*. The start codon for *nad6* was TTA in *Y. paquini* and ATT in the other three spider species. *Trogloneta* had almost the same start/stop codons of PCGs except for the start codons of *nad2* (ATA in *T. yunnanense* and ATT in *T. yuensis*), the stop codons of *cytb* (TAA in *T. yunnanense* and TAG in *T. yuensis*) and *nad1* (TAA in *T. yunnanense* and TAG in *T. yuensis*) (Table 3). Each pair of stop codons between *Y. kehen* and *Y. paquini* were the same, while they shared different stop codons in *nad6* (ATT in *Y. kehen* and TTA in *Y. paquini*) and *nad1* (ATT in *Y. kehen* and ATC in *Y. paquini*) (Table 3).

In this study, a truncated stop codon (T) was detected as existing in *nad4* and *nad4L* in all four spider species, which was similar to the posttranscriptional animals as numerous studies have reported [36,37], *Ebrechtella tricuspidate* [38], *Tetragnatha maxillosa* [34], *Tetragnatha nitens* [34] and *Argiope perforate* [39]. It was assumed that these incomplete stop codons were complemented by posttranscriptional polyadenylation [40]. Additionally, more than 10 bp overlaps were detected in the junctions between *atp8* and *atp6* in each spider mitogenome. Generally, hairpin structures at the 3’ end of the upstream protein’s mRNA may act as a signal for the cleavage of the polycistronic primary transcript. Both truncated stop codon and overlaps between genes indicated selective pressure power to reduce mitochondrial gene size.

**Figure 2:**
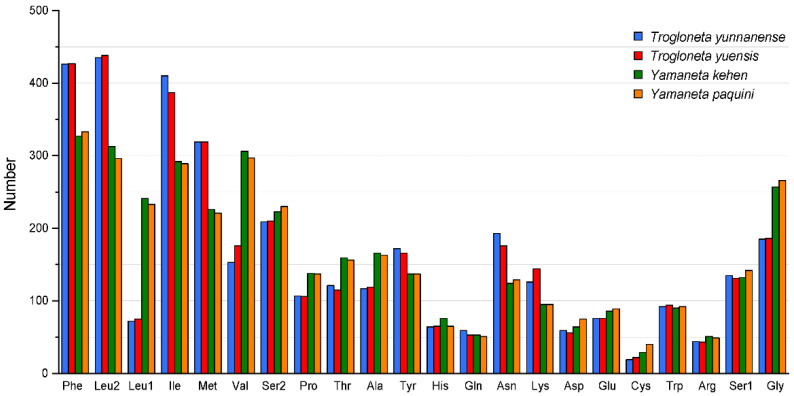
Amino acid frequency of PCGs in the *Trogloneta* and *Yamaneta* mitogenomes.

**Figure 3:**
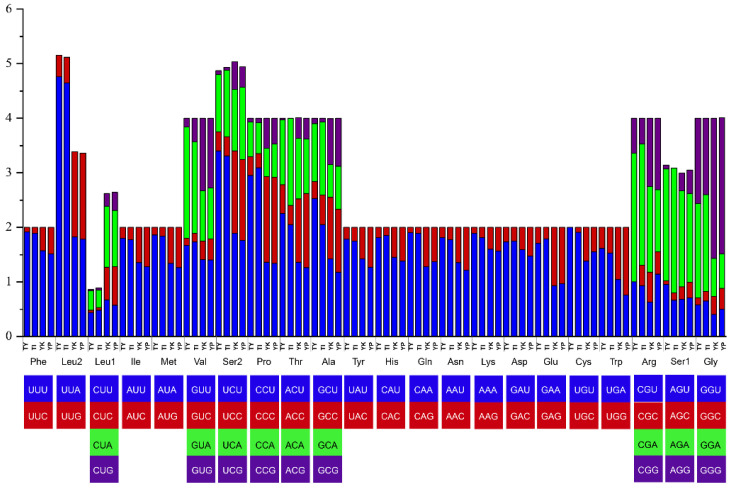
Relative synonymous codon usage of PCGs in the *Trogloneta* and *Yamaneta* mitogenomes. TY, *Trogloneta yunnanense*; TI, *Trogloneta yuensis*; YK, *Yamaneta kehen*; YP, *Yamaneta paquini*.

The total numbers of non-stop codons in *T. yunnanense*, *T. yuensis*, *Y. kehen* and *Y. paquini* were 3593, 3584, 3585 and 3634, respectively. Amino acid frequencies varied significantly between genera but not within genus. The most frequently used amino acids occurred on the leucine (the UUR codon) (mean value = 12.17%) in *Trogloneta* and the phenylalanine (the UUY codon) (mean value = 9.21%) in *Yamaneta* (Figure 2). Cysteine was the least used amino acid in all four spider mitogenomes (mean value = 0.77%). *Trogloneta* contained more codons for Phe, Leu2, Ile, Met, Tyr, Asn and Lys than *Yamaneta*, while Leu1, Val, Ser2, Pro, Thr, Ala, Asp, Glu, Cys and Gly encoded fewer (Figure 2). Analysis of the relative synonymous codon usage (RSCU) revealed the biased usage of A/T rather than G/C at the third codon position. *Yamaneta* used C/G as the third codon more frequently than *Trogloneta* (Figure 3).

To explore the selection pressure between species with different habits, we calculated Ka, Ks and Ka/Ks values within genera. The Ka values varied between 0.017 and 0.107 in *Yamaneta*. Compared to *Yamaneta*, the Ka values displayed relatively higher ranges (0.043–0.346) in *Trogloneta*, Among the 13 mitochondrial PCGs, *atp8* had the biggest Ka/Ks values (0.167 in *Yamaneta* and 0.835 in *Trogloneta*). By contrast, the lowest was cox1 (0.054 in *Yamaneta* and 0.116 in *Trogloneta*) (Table 5).

Mitochondria play a key role in energy metabolism. Previous studies have found that cave spiders and ground spiders have different metabolic rates [41]. In this study, the Ka/Ks values of all 13 PCGs were less than 1, which means they are all under purifying selection. However, compared to *Yamaneta*, *Trogloneta* has much higher Ka/Ks values, which may imply that the effective population size is small in the cave population affected by habitats changes.

### 3.3. Transfer RNA and Ribosomal RNA Gene

It has been widely accepted that the cloverleaf secondary structure of transfer RNA (tRNA) is one of the most conserved features for the mitogenome since the first extremely truncated tRNAs were found in the jumping spider *H. oregonensis* [33]. However, almost all reported spider species contain atypical tRNA secondary structures. A lost-arm tRNA in Arachnids compared to other metazoans or arthropods has been reported more often [42]. It was indicated that the genome-wide propensity to lose sequences that encode canonical cloverleaf structures likely evolved multiple times within arachnids [43]. Given the otherwise extreme conservation of tRNA structure across all of life, one hypothesis was that it was the result of parallel evolution under the pressure of selection pressure [44]. Meanwhile, a posttranscriptional editing mechanism likely edited spider mitogenome tRNA acceptor stems to enable them to function [45]. Thus, these tRNAs with truncated arms could be valuable markers for deep-level phylogenetic inference.

**Figure 4:**
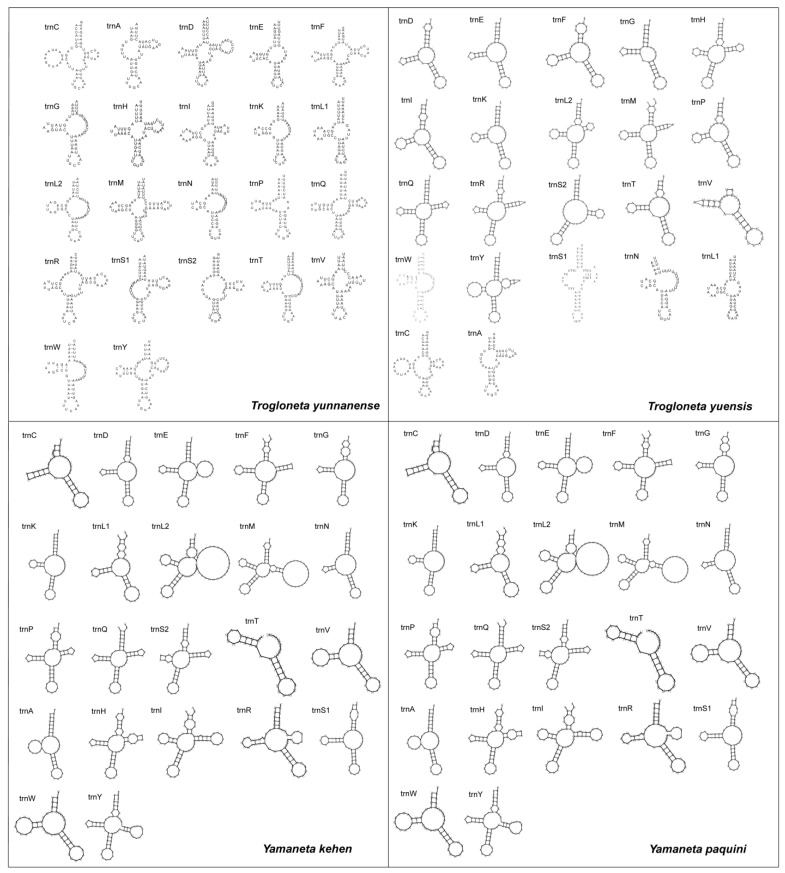
Cloverleaf structure of the 22 inferred tRNAs in the mitogenomes of *Trogloneta* and *Yamaneta* mitogenomes.

For these 22 typical animal tRNA genes in each Mysmenidae mitogenome, 14 tRNAs were encoded by the J-strand and the remaining eight were located on the N-strand, ranging from 37 bp (*trnV* in *T. yuensis*) to 70 bp (*trnI* in *Y. kehen* and *Y. paquini*). Most of the tRNAs in *Trogloneta* and *Yamaneta* mitogenomes had aberrant cloverleaf secondary structures, including a truncated aminoacyl acceptor stem and mismatched (lacking well-paired) aminoacyl acceptor stem (Figure 4). The tRNAs’ secondary structure exhibited a conservative type in *Yamaneta*. All *Yamaneta* tRNAs possess a anticodon arm, and 11 pairs of the same tRNAs (*trnC*, *trnD*, *trnG*, *trnK*, *trnL1*, *trnN*, *trnI*, *trnV*, *trnA*, *trnS1* and *trnW*) lack a TΨC (pseudouracil) arm. However, in *Trogloneta,* six of the twenty-two tRNAs share different secondary structures (*trnD*, *trnG*, *trnF*, *trnI*, *trnL1* and *trnV*), and a DHU (dihydrouracil) arm was absent in three tRNAs (*trnA*, *trnS1* and *trnS2*) of both *T. yunnanense* and *T. yuensis* (Figure 4). A TΨC arm was absent in nine tRNAs of *T. yunnanense* and twelve tRNAs of *T. yuensis*. Our results demonstrated a large divergence in the secondary structure of tRNAs at the genus level, and only four tRNAs (*trnG*, *trnK*, *trnN* and *trnW*) shared the common absence of a TΨC arm among these four spider species. (Figure 4), suggesting a high degree of variability between the two genera.

Previous studies found that *trnS2* (UCN) and *trnS1* (AGN) lacked the DHU arm in many arthropod mitochondrial genomes [46]. Wolstenholme [47] once described that the gene coding for the DHU arm of *trnS1* had been absent prior to metazoan diversification. However, we found that the *trnS1* in *Yamaneta* has an intact secondary structure (Figure 4). The typical cloverleaf structure was also found in *Adoxophyes honmai and Pseudocellus pearsei* [48,49]. The absence of the TψC arm or DHU arm resulted in shorter tRNA gene lengths and more compact gene structures. Lavrov [45] suggested that RNA editing mechanisms may play a key role in posttranscriptional editing to modify these atypical tRNAs.

Large and small subunit rRNAs (*rrnL* and *rrnS*) were adjacent on the N-strand and spaced by a single tRNA (*trnV*). Yet it was hard to accurately predict the ends of rRNAs using DNA sequencing alone. We assumed that the ends of the rRNAs extended to the boundaries of the flanking genes [34]. The length of the predicted rRNAs of these four spider species did not differ much. The length of *rrnL* ranged from 1001 bp in *T. yunnanense* to 1043 bp in *Y. kehen*, and the length of *rrnS* ranged from 686 bp in *T. yunnanense* to 692 bp in *T. yuensis* and *Y. kehen*. The A + T contents of rRNAs were distinct between the two genera. In *Trogloneta,* the A + T contents were 83.8% in *T. yunnanense* and 83.5% in *T. yuensis*; however, in *Yamaneta*, they were 66.9% in *Y. kehen* and 66.5% in *Y. paquini*. 

### 3.4. Control Region

The putative control region located between *rrnS* and *rrnL* was the longest non-coding region in the whole mitogenome. It played a role in initiating and regulating replication and transcription in mitochondria. The full lengths of the CR in the four mitogenomes were 631 bp (*T. yunnanense*), 317 bp (*T. yuensis*), 591 bp (*Y. kehen*) and 581 bp (*Y. paquini*), respectively. The A + T content of the control region of the *Trogloneta* and *Yamaneta* mitogenomes was AT-rich (Table 2), with negative AT skewness value in *T. yunnanense* and positive values in *T. yuensis*, *Y. kehen* and *Y. paquini*. The GC skewness value was positive in all four spider mitogenomes.

### 3.5. Phylogenetic Analysis

**Figure 5:**
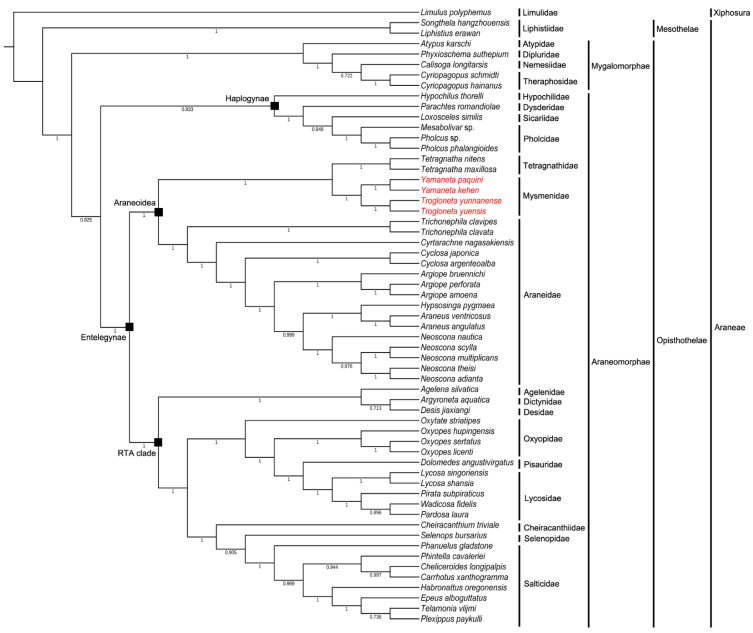
Phylogenetic tree from Araneae species based on nucleotide sequence of 13 PCGs and 2 rRNAs using Bayesian inference (BI). *Limulus polyphemus* was used as an outgroup. Numbers below the nodes refer to Bayesian posterior probabilities in percentages. The specimen used in our experiment are marked in red.

The results of DAMBE and the best-fit models of partitioned analyses can be seen in Figure S1 and Table S1. Almost identical topologies of phylogenetic trees were obtained by the BI and ML methods (Figure 5 and Figure S2). It was clearly observed that the two genera (*Trogloneta* and *Yamaneta*) were clustered into two adjacent clades. The Mysmenidae is a sister group to the Tetragnathidae with bootstrap = 87 and posterior probabilities = 1.00, indicating that Mysmenidae is a member of the superfamily Araneoidea. This evolutionary structure is consistent with earlier studies that used morphological and gene sequence data [50]. In addition, the superfamily Araneoidea was confirmed to be monophyly according to the phylogenetic analysis. The Hypochilidae, which is regarded as an ancient and special taxon in spiders, was also isolated from the families Dysdeidae, Sicariidae and Pholcidae. The RTA clade, containing nine families in this analysis, showed high supporting values for monophyly. However, there were some branches in the RTA clade that were not well supported, such as *Telamonia vlijmi* and *Plexippus paykulli* (bootstrap = 57 and posterior probabilities = 0.736). In this study, two infraorder Mygalomorphae and Araneomopha were clearly split, as inferred from both BI and ML analyses. Moreover, the results strongly supported the monophyletic characteristic of two suborders (Opisthothelae and Mesothelae) in Araneae, with bootstrap = 100 and posterior probabilities = 1.00.

## 4. Conclusions

In the present study, the complete mitogenomes of *T. yunnanense*, *T. yuensis*, *Y. kehen* and *Y. paquini* were determined and characterized. Each mitogenome contained an identical composition, gene order and relatively shorter length compared with most spider mitogenomes. However, the mitogenomes of *Trogloneta* and *Yamaneta* showed high variability. The overall lengths of the *Yamaneta* mitogenomes were relatively longer. They possessed an extremely large spacer about 90 bp (between *trnN* and *trnA*), which had never been found in previous studies. The content of A + T in *Trogloneta* was significantly higher than that in *Yamaneta* among each gene. In addition, the amino acid frequencies of the PCGs and secondary structures of tRNAs between the two genera were significantly different. We found that the mitogenomes of *Yamaneta* were more conserved, especially for the secondary structures of tRNAs that were regarded as markers for deep-level phylogenetic inference. *Y. kehen* and *Y. paquini* contained almost identical truncated arms of tRNAs. The *trnS1* of *Y. kehen* and *Y. paquini* had an intact secondary structure, which was a very rare phenomenon in other species. In *Trogloneta*, on the other hand, six of the twenty-two tRNAs shared different secondary structures. Moreover, the spacers and overlaps were more even between *Y. kehen* and *Y. paquini*, and they also possessed more similar start/stop codons for PCGs than *T. yunnanense* and *T. yuensis.* All of the above evidence supports a higher degree of genetic divergence between *T. yunnanense* and *T. yuensis.* For phylogenetic analysis, the mitogenome revealed the lineage of Araneae with high quality. Our studies indicate that the mitogenomes possess the potential for better exploration of Araneae, and to obtain the sites that are hard to reach with morphological or other molecular methods.

## Figures and Tables

**Table 1:** Information of spider samples and localities.

Species	Sites (Abbrs.)	Geographic Coordinates	Collection Localities
*Trogloneta yunnanense*	Guanniu Cave	27.61372° N, 106.96910° E	Guizhou: Zunyi City, Shenxi Town
*Trogloneta yuensis*	Yuelu Mountain	28.18685° N, 112.94210° E	Hunan: Changsha City, Yuelu Dist.
*Yamaneta kehen*	An anonymous cave	27.12818° N, 098.86014° E	Yunnan: Fugong County, Shiyueliang Town
*Yamaneta paquini*	Walayaku Cave	26.13198° N, 098.86149° E	Yunnan: Lushui County, Daxingdi Town

**Table 2:** Summary of the mitogenomes used for phylogenetic analyses.

Order	Family	Species	Tolal Length (bp)	Total A + T(%)	Accession Number
Araneae	Agelenidae	1. *Agelena silvatica*	14,776	74.5	KX290739.1
	Araneidae	2. *Araneus angulatus*	14,205	75.1	KU365988.1
	Araneidae	3. *Araneus ventricosus*	14,617	73.3	KM588668.1
	Araneidae	4. *Argiope amoena*	14,121	72.1	KJ607907.1
	Araneidae	5. *Argiope bruennichi*	14,063	73.4	KJ594561.1
	Araneidae	6. *Argiope perforata*	14,032	74.2	MK512574.1
	Araneidae	7. *Cyclosa argenteoalba*	14,575	73.7	KP862583.1
	Araneidae	8. *Cyclosa japonica*	14,687	72.9	MK512575.1
	Araneidae	9. *Cyrtarachne nagasakiensis*	14,402	75.7	KR259802.1
	Araneidae	10. *Hypsosinga pygmaea*	14,193	76.1	KR259803.1
	Araneidae	11. *Neoscona adianta*	14,161	74.6	KR259805.1
	Araneidae	12. *Neoscona multiplicans*	14,074	74.8	MK052682.1
	Araneidae	13. *Neoscona nautica*	14,049	78.8	KR259804.1
	Araneidae	14. *Neoscona scylla*	14,092	74.7	MK086023.1
	Araneidae	15. *Neoscona theisi*	14,156	75.2	KP100667.1
	Atypidae	16. *Atypus karschi*	14,149	73.6	MT832081.1
	Cheiracanthiidae	17. *Cheiracanthium triviale*	14,595	77.9	MN334527.1
	Desidae	18. *Desis jiaxiangi*	14,610	77.1	MW178198.1
	Dictynidae	19. *Argyroneta aquatica*	16,000	71.9	KJ907736.1
	Dipluridae	20. *Phyxioschema suthepium*	13,931	67.4	JQ407802.1
	Dysderidae	21. *Parachtes romandiolae*	14,220	71.4	MN052923.1
	Hypochilidae	22. *Hypochilus thorelli*	13,991	70.3	EU523753.1
	Liphistiidae	23. *Liphistius erawan*	14,197	67.7	JQ407803.1
	Liphistiidae	24. *Songthela hangzhouensis*	14,215	72.2	AY309258.1
	Lycosidae	25. *Lycosa shansia*	14,638	79.2	OK032619.1
	Lycosidae	26. *Lycosa singoriensis*	13,686	75.1	OK032620.1
	Lycosidae	27. *Pardosa laura*	14,513	77.4	KM272948.1
	Lycosidae	28. *Pirata subpiraticus*	14,528	75.6	KM486623.1
	Lycosidae	29. *Wadicosa fidelis*	14,741	76.1	KP100666.1
	Mysmenidae	30. *Trogloneta yuensis*	13,771	78.7	ON093239
	Mysmenidae	31. *Trogloneta yunnanense*	14,103	79.6	ON093232
	Mysmenidae	32. *Yamaneta kehen*	14,223	63.8	OP413741
	Mysmenidae	33. *Yamaneta paquini*	14,208	63	OP413742
	Nemesiidae	34. *Calisoga longitarsis*	14,070	64	EU523754.1
	Nephilidae	35. *Trichonephila clavata*	14,436	76	AY452691.1
	Nephilidae	36. *Trichonephila clavipes*	14,902	77.2	LC619787.1
	Oxyopidae	37. *Oxyopes hupingensis*	15,078	77.9	MK518391.1
	Oxyopidae	38. *Oxyopes licenti*	14,431	78.1	MT741489.1
	Oxyopidae	39. *Oxyopes sertatus*	14,442	75.9	KM272950.1
	Pholcidae	40. *Mesabolivar* sp.	14,941	70.6	MH643812.1
	Pholcidae	41. *Pholcus phalangioides*	14,459	65.8	JQ407804.1
	Pholcidae	42. *Pholcus* sp.	14,279	65.8	KJ782458.1
	Pisauridae	43. *Dolomedes angustivirgatus*	14,783	76.8	KU354434.1
	Salticidae	44. *Carrhotus xanthogramma*	14,563	75.1	KP402247.1
	Salticidae	45. *Cheliceroides longipalpis*	14,334	79	MH891570.1
	Salticidae	46. *Epeus alboguttatus*	14,625	77.6	MH922026.1
	Salticidae	47. *Habronattus oregonensis*	14,381	74.3	AY571145.1
	Salticidae	48. *Phanuelus gladstone*	14,458	75.1	MT773150.1
	Salticidae	49. *Phintella cavaleriei*	14,325	78.1	MW540530.1
	Salticidae	50. *Plexippus paykulli*	14,316	73.5	KM114572.1
	Salticidae	51. *Telamonia vlijmi*	14,601	77.3	KJ598073.1
	Selenopidae	52. *Selenops bursarius*	14,272	74.4	KM114573.1
	Sicariidae	53. *Loxosceles similis*	14,683	72.8	MK425700.1
	Tetragnathidae	54. *Tetragnatha maxillosa*	14,578	74.5	KP306789.1
	Tetragnathidae	55. *Tetragnatha nitens*	14,639	74.3	KP306790.1
	Theraphosidae	56. *Cyriopagopus hainanus*	13,874	69.6	MN877932.1
	Theraphosidae	57. *Cyriopagopus schmidti*	13,874	69.8	AY309259.1
	Thomisidae	58. *Oxytate striatipes*	14,407	78.2	KM507783.1
Xiphosura	Limulidae	59. *Limulus polyphemus*	14,985	67.6	NC003057.1

**Table 3:** Gene order and features of mitochondrial genome of four Mysmenidae species.

Gene	Size	Size	Size	Size	Intergenic Sequence	Start/Stop Codons
	TY	TI	YK	YP	TY; TI; YK; YP	TY; TI; YK; YP
*trnM*(cat)	66	61	76	76		
*nad2*	912	930	918	921	0; −12; 0; −3	ATA/TAA; ATT/TAA; ATT/TAG; ATT/TAG
*trnW*(tca)	48	48	53	53	+6; +6; −5; −5	
*trnY*(gta)	64	64	67	67	–32; −33; −25; −25	
*trnC*(gca)	63	61	48	48	−27; −23; −6; −6	
*cox1*	1524	1524	1536	1536	−1; −1; −5; −5	TTA/TAA; TTA/TAA; TTA/TAA; TTA/TAA
*cox2*	666	666	660	660	+3; +3; +9; +9	TTG/TAA; TTG/TAA; ATT/TAG; ATT/TAG
*trnK*(ctt)	52	54	62	62	0; −2; −2; −2	
*trnD*(gtc)	68	51	58	62	−12; −10; −18; −18	
*atp8*	123	150	150	150	+6; −4; −11; −15	ATT/TAA; ATT/TAA; ATC/TAG; ATC/TAG
*atp6*	669	672	672	672	−10; −13; −13; −13	ATG/TAA; ATG/TAA; ATG/TAA; ATG/TAA
*cox3*	786	783	810	810	+3; +3; +5; +4	TTG/TAG; TTG/TAG; TTG/TAA; TTG/TAA
*trnG*(tcc)	54	47	60	52	−2; −22; −26; −25	
*nad3*	336	336	336	336	−7; −3; −13; −5	ATA/TAG; ATA/TAG; ATT/TAA; ATT/TAA
*trnL2*(taa)	56	62	49	49	−15; −19; −6; −6	
*trnN*(gtt)	65	55	55	54	−3; −9; −3; −3	
*trnA*(tgc)	48	54	55	55	−11; +5; +89; +87	
*trnS1*(tct)	60	60	59	59	−8; −3; −10; −10	
*trnR*(tcg)	66	63	62	62	−7; −5; +1; +2	
*trnE*(ttc)	51	54	64	65	−31; −28; −16; −16	
*trnF*(gaa)	67	55	65	59	−26; −19; −34; −28	
*nad5*	1632	1629	1641	1641	−5; −5; −2; −1	ATA/TAA; ATA/TAA; ATA/TAA; ATA/TAA
*trnH*(gtg)	58	63	62	62	+8; −17; −7; −8	
*nad4*	1282	1273	1270	1270	−2; +6; −3; −3	ATA/T; ATA/T; ATA/T; ATA/T
*nad4L*	262	262	265	265	+3; +5; +3; +3	ATT/T; ATT/T; ATT/T; ATT/T
*trnP*(tgg)	48	51	59	65	+6; +2; −12; −16	
*nad6*	420	435	468	474	+10; +10; −8; −16	ATT/TAA; ATT/TAA; ATT/TAA; TTA/TAA
*trnI*(gat)	59	51	70	70	−3; −17; −26; −26	
*cytb*	1140	1152	1146	1146	−3; −9; −27; −28	ATG/TAA; ATG/TAG; ATG/TAG; ATG/TAG
*trnS2*(tga)	55	56	62	62	−11; −11; −16; −16	
*trnT*(tgt)	61	54	43	43	−6; −4; +7; +7	
*nad1*	921	918	921	921	−17; −12; −6; −6	ATT/TAA; ATT/TAG; ATT/TAG; ATC/TAG
*trnL1*(tag)	51	61	54	54	−3; −13; −4; −4	
*rrnL*	1001	1016	1043	1040	0; 0; 0; 0	
*trnV*(tac)	53	37	52	52	0; 0; 0; 0	
*rrnS*	686	692	692	691	0; 0; 0; 0	
*trnQ*(ttg)	64	62	61	61	0; 0; 0; 0	
CR	631	317	591	581	0; 0; 0; 0	

Note: TY, Trogloneta yunnanense; TI, Trogloneta yuensis; YK, Yamaneta kehen; YP, Yamaneta paquini.

**Table 4:** A + T content (%), AT and GC skewness of four Mysmenidae species mitogenomes.

Regions	Strand	AT (%)				AT Skew				GC Skew			
		TY	TI	YK	YP	TY	TI	YK	YP	TY	TI	YK	YP
Full genome	+	79.6	78.7	63.8	63	−0.068	−0.054	−0.168	−0.174	0.428	0.288	0.428	0.421
PCGs	+	77.8	77.2	62.1	61.2	−0.203	−0.189	−0.342	−0.343	0.444	0.331	0.444	0.437
PCGs	−	79.4	78.8	62.5	61.6	−0.119	−0.131	0	0.011	−0.514	−0.339	−0.514	−0.504
tRNAs	+	81.9	80.2	71.9	71.5	−0.031	−0.060	−0.072	−0.063	0.378	0.236	0.378	0.386
tRNAs	−	81.7	81.3	74.4	73.4	0.016	−0.250	0.067	0.098	−0.068	0.014	−0.068	−0.103
rRNAs	−	83.8	83.5	66.9	66.5	0.027	0.006	0.087	0.109	−0.341	−0.050	−0.341	−0.338
1st codon position	+	71.6	70.8	57.8	58.4	−0.061	−0.065	−0.212	−0.219	0.463	0.420	0.463	0.503
1st codon position	−	78	77.8	63.7	63.6	0.033	0.008	0.197	0.201	−0.234	−0.013	−0.234	−0.226
2nd codon position	+	71.2	71.2	65.6	64.6	−0.406	−0.401	−0.443	−0.423	0.134	0.109	0.134	0.169
2nd codon position	−	71.9	71.9	64.9	64.6	−0.413	−0.450	−0.463	−0.456	−0.481	−0.371	−0.481	−0.478
3rd codon position	+	90.6	89.6	63.2	60.6	−0.155	−0.118	−0.357	−0.376	0.710	0.691	0.710	0.609
3rd codon position	−	88.4	86.8	58.8	56.5	−0.013	0.008	0.300	0.331	−0.790	−0.822	−0.790	−0.758
Control region	+	84.8	81.7	72.4	71.2	−0.065	0.004	0.103	0.155	0.125	0.069	0.141	0.066

Note: TY, Trogloneta yunnanense; TI, Trogloneta yuensis; YK, Yamaneta kehen; YP, Yamaneta paquini.

**Table 5:** Ka, Ks and Ka/Ks values for 13 PCGs of *Trogloneta and Yamaneta*.

	*Trogloneta*			*Yamaneta*		
Gene	Ka	Ks	Ka/Ks	Ka	Ks	Ka/Ks
*atp6*	0.158	0.314	0.505	0.053	0.490	0.108
*atp8*	0.346	0.415	0.835	0.107	0.641	0.167
*cox1*	0.043	0.373	0.116	0.017	0.323	0.054
*cox2*	0.093	0.319	0.292	0.041	0.436	0.094
*cox3*	0.154	0.423	0.363	0.054	0.331	0.162
*cytb*	0.186	0.296	0.628	0.033	0.409	0.081
*nad1*	0.113	0.703	0.161	0.036	0.420	0.085
*nad2*	0.173	0.358	0.484	0.057	0.546	0.103
*nad3*	0.216	0.329	0.657	0.066	0.452	0.146
*nad4*	0.138	0.591	0.233	0.052	0.328	0.157
*nad4L*	0.159	0.620	0.256	0.033	0.464	0.071
*nad5*	0.157	0.732	0.214	0.046	0.416	0.110
*nad6*	0.263	0.403	0.651	0.062	0.313	0.199

## Data Availability

The complete mitochondrial genomes generated in this study have been deposited in GenBank with accession numbers listed in Table 2.

## Supplementary Materials

The following supporting information can be downloaded at: https://www.mdpi.com/article/10.3390/ani13030497/s1, Figure S1: The result of substitution saturation test by using DAMBE; Figure S2: The ML phylogenetic tree from Araneae species based on nucleotide sequence of 13 PCGs and 2 rRNAs; Table S1: The best-fit models of each partition

## References

[1] Li M., Chen W.T., Zhang Q.L., Liu M., Xing C.W., Cao Y., Luo F.Z., Yuan M.L. (2022). Mitochondrial phylogenomics provides insights into the phylogeny and evolution of spiders (Arthropoda: Araneae). Zool. Res..

[2] Coddington J.A., Levi H.W. (1991). Systematics and Evolution of Spiders (Araneae). Annu. Rev. Ecol. Syst..

[3] World Spider Catalog Version 23.5. Natural History Museum Bern. http://wsc.nmbe.ch.

[4] Lopardo L., Giribet G., Hormiga G. (2011). Morphology to the rescue: Molecular data and the signal of morphological characters in combined phylogenetic analyses-a case study from mysmenid spiders (Araneae, Mysmenidae), with comments on the evolution of web architecture. Cladistics.

[5] Arnason U., Gullberg A., Gretarsdottir S., Ursing B., Janke A. (2000). The Mitochondrial Genome of the Sperm Whale and a New Molecular Reference for Estimating Eutherian Divergence Dates. J. Mol. Evol..

[6] Feng C.C., Miller J.A., Lin Y.C., Shu Y.F. (2019). Further study of two Chinese cave spiders (Araneae, Mysmenidae), with description of a new genus. Zookeys.

[7] Garrison N.L., Rodriguez J., Agnarsson I., Coddington J.A., Griswold C.E., Hamilton C.A., Hedin M., Kocot K.M., Ledford J.M., Bond J.E. (2016). Spider phylogenomics: Untangling the Spider Tree of Life. PeerJ.

[8] Grechko V.V. (2002). Using molecular DNA markers in phylogeny and systematics. Genetika.

[9] Boore J.L. (1999). Animal mitochondrial genomes. Nucleic Acids Res..

[10] Zhu Z.M., Gao X.F. (2015). Molecular evidence for the hybrid origin of Rosa lichiangensis (Rosaceae). Phytotaxa.

[11] Lopardo L., Hormiga G. (2015). Out of the twilight zone: Phylogeny and evolutionary morphology of the orb-weaving spider family Mysmenidae, with a focus on spinneret spigot morphology in symphytognathoids (Araneae, Araneoidea). Zool. J. Linn. Soc..

[12] Howarth F.G. (1983). Ecology of Cave Arthropods. Annu. Rev. Entomol..

[13] Mammola S., Isaia M. (2017). Spiders in caves. Proc. R. Soc. B Biol. Sci..

[14] Song D.X., Zhu M.S., Chen Y.Z. (1994). On some species of cave arachnids of China. Sixtieth Anniversary of the Founding of China Zoological Society: Memorial Volume Dedicated to the Hundredth Anniversary of the Birthday of the Late Prof. Sisan Chen (Z. Chen).

[15] Miller J.A., Griswold C.E., Yin C.M. (2009). The symphytognathoid spiders of the Gaoligongshan, Yunnan, China (Araneae, Araneoidea): Systematics and diversity of micro-orbweavers. Zookeys.

[16] Chen S.F., Zhou Y.Q., Chen Y.R., Gu J. (2018). fastp: An ultra-fast all-in-one FASTQ preprocessor. Bioinformatics.

[17] Dierckxsens N., Mardulyn P., Smits G. (2017). NOVOPlasty: De novo assembly of organelle genomes from whole genome data. Nucleic Acids Res..

[18] Bernt M., Donath A., Jühling F., Externbrink F., Florentz C., Fritzsch G., Pütz J., Middendorf M., Stadler P.F. (2013). MITOS: Improved de novo metazoan mitochondrial genome annotation. Mol. Phylogenetics Evol..

[19] Altschul S., Gish W., Miller W., Myers E.W., Lipman D.J. (1990). Basic local alignment search tool. J. Mol. Biol..

[20] Greiner S., Lehwark P., Bock R. (2019). OrganellarGenomeDRAW (OGDRAW) version 1.3.1: Expanded toolkit for the graphical visualization of organellar genomes. Nucleic Acids Res..

[21] Lowe T.M., Chan P.P. (2016). tRNAscan-SE On-line: Integrating search and context for analysis of transfer RNA genes. Nucleic Acids Res..

[22] Laslett D., Canback B. (2008). ARWEN: A program to detect tRNA genes in metazoan mitochondrial nucleotide sequences. Bioinformatics.

[23] Kumar S., Stecher G., Li M., Knyaz C., Tamura K. (2018). MEGA X: Molecular Evolutionary Genetics Analysis across Computing Platforms. Mol. Biol. Evol..

[24] Perna N.T., Kocher T.D. (1995). Patterns of Nucleotide Composition at Fourfold Degenerate Sites of Animal Mitochondrial Genomes. J. Mol. Evol..

[25] Rozas J., Ferrer-Mata A., Sanchez-DelBarrio J.C., Guirao-Rico S., Librado P., Ramos-Onsins S.E., Sanchez-Gracia A. (2017). DnaSP 6: DNA Sequence Polymorphism Analysis of Large Data Sets. Mol. Biol. Evol..

[26] Lavrov D.V., Boore J.L., Brown W.M. (2000). The complete mitochondrial DNA sequence of the horseshoe crab Limulus polyphemus. Mol. Biol. Evol..

[27] Castresana J. (2000). Selection of conserved blocks from multiple alignments for their use in phylogenetic analysis. Mol. Biol. Evol..

[28] Xia X., Xie Z. (2001). DAMBE: Software package for data analysis in molecular biology and evolution. J. Hered..

[29] Kalyaanamoorthy S., Minh B.Q., Wong T.K.F., von Haeseler A., Jermiin L.S. (2017). ModelFinder: Fast model selection for accurate phylogenetic estimates. Nat. Methods.

[30] Ronquist F., Teslenko M., van der Mark P., Ayres D.L., Darling A., Hohna S., Larget B., Liu L., Suchard M.A., Huelsenbeck J.P. (2012). MrBayes 3.2: Efficient Bayesian Phylogenetic Inference and Model Choice Across a Large Model Space. Syst. Biol..

[31] Nguyen L.T., Schmidt H.A., von Haeseler A., Minh B.Q. (2015). IQ-TREE: A Fast and Effective Stochastic Algorithm for Estimating Maximum-Likelihood Phylogenies. Mol. Biol. Evol..

[32] Zhang D., Gao F.L., Jakovlic I., Zou H., Zhang J., Li W.X., Wang G.T. (2020). PhyloSuite: An integrated and scalable desktop platform for streamlined molecular sequence data management and evolutionary phylogenetics studies. Mol. Ecol. Resour..

[33] Masta S.E., Boore J.L. (2004). The complete mitochondrial genome sequence of the spider Habronattus oregonensis reveals rearranged and extremely truncated tRNAs. Mol. Biol. Evol..

[34] Wang Z.L., Li C., Fang W.Y., Yu X.P. (2016). The Complete Mitochondrial Genome of two Tetragnatha Spiders (Araneae: Tetragnathidae): Severe Truncation of tRNAs and Novel Gene Rearrangements in Araneae. Int. J. Biol. Sci..

[35] Xu K.K., Lin X.C., Yang D.X., Yang W.J., Li C. (2019). Characterization of the complete mitochondrial genome sequence of Neoscona scylla and phylogenetic analysis. Mitochondrial DNA Part B Resour..

[36] Kumar V., Tyagi K., Chakraborty R., Prasad P., Kundu S., Tyagi I., Chandra K. (2020). The Complete Mitochondrial Genome of endemic giant tarantula, Lyrognathus crotalus (Araneae: Theraphosidae) and comparative analysis. Sci. Rep..

[37] Sheffield N.C., Hiatt K.D., Valentine M.C., Song H.J., Whiting M.F. (2010). Mitochondrial genomics in Orthoptera using MOSAS. Mitochondrial DNA.

[38] Zhu H.F., Wang Z.Y., Wang Z.L., Yu X.P. (2019). Complete mitochodrial genome of the crab spider Ebrechtella tricuspidata (Araneae: Thomisidae): A novel tRNA rearrangement and phylogenetic implications for Araneae. Genomics.

[39] Yang W.J., Xu K.K., Liu Y., Yang D.X., Li C. (2019). Complete mitochondrial genome and phylogenetic analysis of Argiope perforata (Araneae: Araneidae). Mitochondrial DNA Part B Resour..

[40] Ojala D., Montoya J., Attardi G. (1981). Transfer-Rna Punctuation Model of RNA Processing in Human Mitochondria. Nature.

[41] Deelemanreinhold C.L. (1978). Spiders of Genus Rhode in Yugoslavia (Araneae, Dysderidae). Int. J. Speleol..

[42] Pons J., Bover P., Bidegaray-Batista L., Arnedo M.A. (2019). Arm-less mitochondrial tRNAs conserved for over 30 millions of years in spiders. BMC Genom..

[43] Masta S.E., Boore J.L. (2008). Parallel evolution of truncated transfer RNA genes in arachnid mitochondrial genomes. Mol. Biol. Evol..

[44] Rand D.M. (1993). Endotherms, Ectotherms, and Mitochondrial Genome-Size Variation. J. Mol. Evol..

[45] Lavrov D.V., Brown W.M., Boore J.L. (2000). A novel type of RNA editing occurs in the mitochondrial tRNAs of the centipede Lithobius forficatus. Proc. Natl. Acad. Sci. USA.

[46] Li C. (2016). Sequencing and Phylogeny Analysis of the Complete Mitogenomes of Six Orb-Weaving Spiders.

[47] Wolstenholme D.R. (1992). Animal Mitochondrial-DNA—Structure and Evolution. Int. Rev. Cytol..

[48] Ronquist F., Huelsenbeck J.P. (2003). MrBayes 3: Bayesian phylogenetic inference under mixed models. Bioinformatics.

[49] Lee E.S., Shin K.S., Kim M.S., Park H., Cho S.W., Kim C.B. (2006). The mitochondrial genome of the smaller tea tortrix Adoxophyes honmai (Lepidoptera: Tortricidae). Gene.

[50] Hormiga G., Griswold C.E. (2014). Systematics, Phylogeny, and Evolution of Orb-Weaving Spiders. Annu. Rev. Entomol..

