# Supplementary material for: New Insights into the Origin and Evolution of Mysmenid Spiders (Araneae, Mysmenidae) Based on the First Four Complete Mitochondrial Genomes
Source: Animals (Basel). 2023 Jan 31;13(3):497. doi: 10.3390/ani13030497 (PMC9913698; doi:10.3390/ani13030497)
Supplement: Supplementary file 1 [file animals-13-00497-s001.zip › Figure S2.pdf]

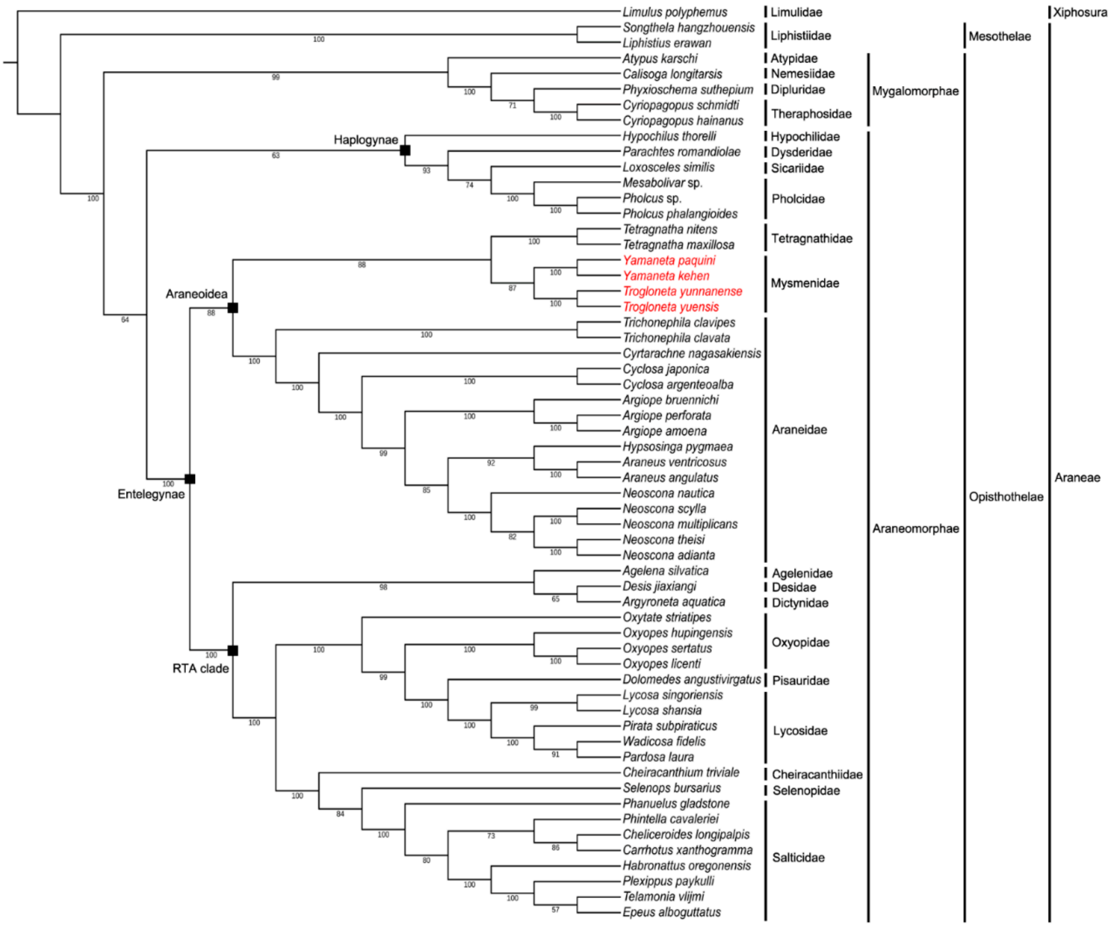

Figure S2. Phylogenetic tree from Araneae species based on nucleotide sequence of 13 PCGs and 2 rRNAs using maximum likelihood (ML). *Limulus polyphemus* was used as an outgroup. Numbers below the nodes referred to ML bootstrap values. The specimen used in our experiment were marked in red.
