# Supplementary figures and images for: New Insights into the Origin and Evolution of Mysmenid Spiders (Araneae, Mysmenidae) Based on the First Four Complete Mitochondrial Genomes
Source: Animals (Basel). 2023 Jan 31;13(3):497. doi: 10.3390/ani13030497 (PMC9913698; doi:10.3390/ani13030497)

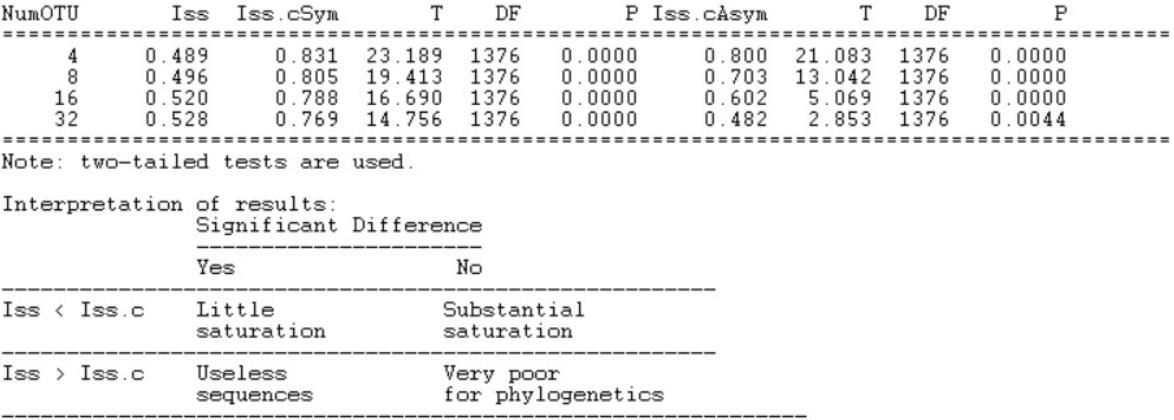

Supplement: Supplementary file 1 [file animals-13-00497-s001.zip › Figure S1.jpg]
